# Morphology Evolution, Molecular Simulation, Electrical Properties, and Rheology of Carbon Nanotube/Polypropylene/Polystyrene Blend Nanocomposites: Effect of Molecular Interaction between Styrene-Butadiene Block Copolymer and Carbon Nanotube

**DOI:** 10.3390/polym13020230

**Published:** 2021-01-11

**Authors:** Ivonne Otero Navas, Milad Kamkar, Mohammad Arjmand, Uttandaraman Sundararaj

**Affiliations:** 1 Department of Chemical and Petroleum Engineering, Schulich School of Engineering, University of Calgary, Calgary, AB T2N 1N4, Canada; imoteron@ucalgary.ca (I.O.N.); milad.kamkar1@ucalgary.ca (M.K.); 2 School of Engineering, University of British Columbia, Kelowna, BC V1V 1V7, Canada; mohammad.arjmand@ubc.ca

**Keywords:** polymer blend, morphology, rheology, LAOS, electrical conductivity

## Abstract

This work studied the impact of three types of styrene-butadiene (SB and SBS) block copolymers on the morphology, electrical, and rheological properties of immiscible blends of polypropylene:polystyrene (PP:PS)/multi-walled carbon nanotubes (MWCNT) with a fixed blend ratio of 70:30 vol.%. The addition of block copolymers to PP:PS/MWCNT blend nanocomposites produced a decrease in the droplet size. MWCNTs, known to induce co-continuity in PP:PS blends, did not interfere with the copolymer migration to the interface and, thus, there was morphology refinement upon addition of the copolymers. Interestingly, the addition of the block copolymers decreased the electrical resistivity of the PP:PS/1.0 vol.% MWCNT system by 5 orders of magnitude (i.e., increase in electrical conductivity). This improvement was attributed to PS Droplets-PP-Copolymer-Micelle assemblies, which accumulated MWCNTs, and formed an integrated network for electrical conduction. Molecular simulation and solubility parameters were used to predict the MWCNT localization in the immiscible blend. The simulation results showed that diblock copolymers favorably interact with the nanotubes in comparison to the triblock copolymer, PP, and PS. However, the interaction between the copolymers and PP or PS is stronger than the interaction of the copolymers and MWCNTs. Hence, the addition of copolymer also changed the localization of MWCNT from PS to PS–PP–Micelles–Interface, as observed by TEM images. In addition, in the last step of this work, we investigated the effect of the addition of copolymers on inter- and intra-cycle viscoelastic behavior of the MWCNT incorporated polymer blends. It was found that addition of the copolymers not only affects the linear viscoelasticity (e.g., increase in the value of the storage modulus) but also dramatically impacts the nonlinear viscoelastic behavior under large deformations (e.g., higher distortion of Lissajous–Bowditch plots).]

## 1. Introduction

**Figure 1:**
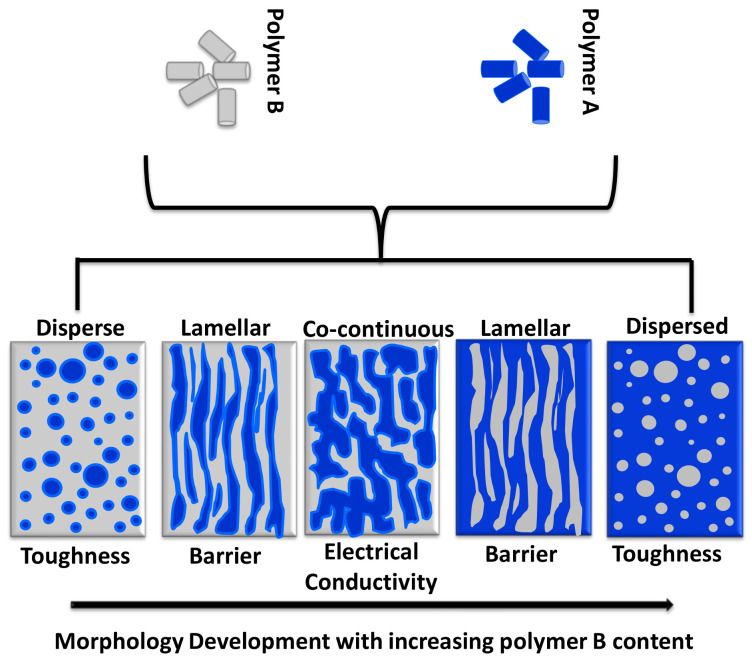
Polymer blend morphologies and their characteristic properties. The image was adapted from reference [13].

Filled polymer blends are among the most attractive options to develop new materials for electrical conduction. The mixture of two or more polymers can be miscible or immiscible. Miscible polymer blends are not common, and most of the polymer blends are immiscible. For immiscible blends, depending on the number of species incorporated, two or more phases form and different morphological structures are generated [1,2,3,4,5]. Due to the considerable impact of the morphology on the final properties of the polymer blends, morphology development has drawn significant attention in both academia and industry communities [1,6,7,8,9]. The generated morphologies are associated with the rheological properties of blend components, the composition of the blend, and interfacial characteristics of the blend system. Depending on the ratio of components in the blends and by controlling different mixing conditions [10,11], diverse structures can be generated (Figure 1): droplets dispersed in a matrix phase, employed to increase the toughness of pristine polymers [12]; fiber-like and lamellar structures, which offer barrier properties [10,13]; co-continuous morphology, suitable for electrical applications [14,15]; and several other structures.

When a conductive nanofiller (CN) is mixed with an immiscible biphasic polymer blend, the nanofiller can be localized in one of the phases, in both phases, and/or at the interphase. This localization is driven by thermodynamic [8,9,16,17] and kinetic [7,18,19] parameters. The selective localization of CNs in immiscible blends can be used as a strategy to tune the final electrical properties of the polymer blend nanocomposite. For instance, if a CN is localized in one of the phases of a co-continuous polymer blend and the amount of CN in that phase is enough to form an electrically conductive network, then a double percolated structure is generated [14,20,21,22,23]. That is, the conductive filler only needs to percolate within the already percolated polymer phase. This particular method has been used as a strategy to decrease the percolation threshold, i.e., the critical amount of CN needed for electrical conduction [14,20,21]. On the other hand, when CNs are localized inside the dispersed phase of an immiscible matrix-dispersed polymer blend, the lack of connectivity of the minor phase (i.e., separated droplets) impedes the electron transport in the material, hence decreasing the current leakage and, thus, making the material suitable for charge storage applications [24].

The addition of CNs into multiphase polymer blends produces morphological changes, such as a decrease or increase in domain size [25,26,27,28] or an increase in co-continuity [24,29,30] in biphasic polymer blends, which affect the final properties and performance of the nanocomposite. However, these structures are generally not in a thermodynamic equilibrium state [31]. Equilibrium cannot be attained in a reasonable time frame due to multiple factors, such as the macromolecular nature of the polymers, which results in high viscosity and increased viscoelastic behavior of these systems, which in turn leads to low mobility rates of the nanofillers in the polymeric matrix [32,33].

Compatibilizers or copolymers are often added to the immiscible polymer blends to stabilize the blend morphology [34,35,36,37], mitigate the system heterogeneity, and improve the phase adhesion between the components. Phase adhesion provides significant improvement in the mechanical properties of immiscible polymer blends [38] which can be detected by rheological approaches. The copolymer localizes at the interphase of the immiscible blend and decreases the coalescence by steric stabilization of the interphase [5,39] and/or the Marangoni stresses [39,40,41]. Marangoni stresses result from the gradient of block copolymer at the interphase and decrease in the rate of the film drainage processes during the coarsening of the droplets. Other authors have reported that the addition of copolymers decreases the interfacial tension [35,42], implying an increase in miscibility of the blend components.

The results presented in this manuscript illustrate how the addition of styrene-butadiene (SB) block copolymer can alter the localization of multi-walled carbon nanotubes (MWCNT) in polypropylene:polystyrene (PP:PS) immiscible blends. This modification with block copolymers concurrently enhances the electrical and rheological properties and decreases the domain size of the polymer blend nanocomposite. It was found that the addition of block copolymers decreased the electrical resistivity and increased the storage modulus by 5 and 1 orders of magnitude, respectively, for PP:PS/70:30/MWCNT 1.0 vol.%. The results were explained using MWCNT selective localization in the blend system and morphological changes (decrease in domain size) upon the addition of block copolymers. To justify the results, molecular simulation and solubility parameters were employed to quantify the interactions between the polymers, copolymer, and MWCNT. Additionally, as the materials experience large and rapid deformation in real processing conditions in industry, we studied the effects of copolymer addition and morphology on inter- and intra-cycle rheological response of the MWCNT incorporated blends under large amplitude oscillatory shear (LAOS) flow. Stress decomposition method was utilized to explain the intra-cycle viscoelastic response of the blends in nonlinear region. It is shown that the stress waveform considerably depends on the type of the copolymer, signifying that Lissajous–Bowditch plots can be used as a strong tool to distinguish the polymer blends.

## 2. Materials and Methods

### 2.1. Gaussian Molecular Simulation

**Figure 2:**
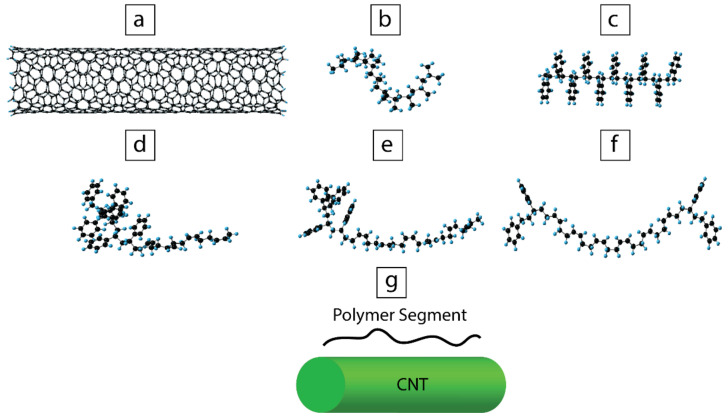
Optimized geometries of (**a**) 5,9-chiral carbon nanotubes (CNT) segment, (**b**) polypropylene (PP) segment (10 repeating units), (**c**) polystyrene (PS) segment (10 repeating units), (**d**) styrene-butadiene (SB) diblock copolymer segment S6B4, (**e**) SB diblock copolymer segment S4B6, (**f**) styrene-butadiene-styrene (SBS) triblock copolymer segment S2B6S2, and (**g**) schematic of the initial configuration of polymer segment/CNT assembly. The green cylinder represents CNT, and polymer segment corresponds to PP, PS, or copolymer.

The molecular simulation was performed using the GAUSSIAN 16 software (Gaussian INC, Wallingford, CT, USA) [43] to understand the interaction between the surface of CNT with polymer segments of PP, PS, styrene-butadiene (SB) diblock copolymer, or styrene-butadiene-styrene (SBS) triblock copolymer. The CNT used in the simulation had a 5,9-chiral structure with an average diameter of 10.5 Å and a length of 53.3 Å. The ends of the simulated CNTs were passivated using hydrogen atoms to avoid end effects (see Figure 2a). PP, PS, diblock, and triblock segments were constituted by 10 repeating units. PP and PS chain segments had isotactic and atactic structures, respectively (Figure 2b,c). Three different segments of linear styrene-butadiene block copolymers were considered: A diblock copolymer with 6 and 4 repeating units of styrene and butadiene, respectively (S6B4) (Figure 2d); a diblock composed of 6 butadiene and 4 styrene repeating units (S4B6) (Figure 2e); and a triblock with 6 butadiene units in the middle, and two styrene segments of 2 repeating units at the sides (S2B6S2) (Figure 2f). The configurations of the copolymers were chosen in such a way to consider the symmetry effect of the styrene and butadiene arrangement in the interaction with CNTs. The initial interaction distance between CNTs and each of the polymer segments was set to 4 Å (Figure 2g).

The simulation process consisted of the geometrical optimization of the CNTs, polymer and copolymer segments, and polymer/CNT and copolymer/CNT assemblies. This optimization was based on the energy minimization, which consisted of changing the spatial arrangement of the atoms of the molecules until the inter-atomic forces at each atom are close to zero. To confirm that the obtained structures corresponded to the global minimum in energy, a frequency calculation (second derivative of the energy with respect to the geometrical coordinates) was performed. All the calculations were performed employing the semi-empirical parameterized model 6 (PM6) by ignoring diatomic differential overlap (NDDO) [44].

To study the interactions between the polymer and copolymer segments and the CNTs, binding energy, and molecular electrostatic potentials were evaluated. The binding energy parameter is defined in Equation (1).

(1) $$\Delta E=E_{CNT}+E_{s}-E_{s/CNT},$$

where $E_{CNT}$, $E_{s}$, $E_{s/CNT}$ correspond to the energy of the optimized structures of the (5,9)-chiral CNT, the energy of the optimized segment structures of PP, PS, S6B4, S4B6, or S2B6S2, and the energy of the optimized structures of polymer/CNT systems, respectively. If the interaction was favorable, positive binding energy was obtained.

### 2.2. Materials and Composites Preparation

Polypropylene (PP) H0500HN (M_w_ = 209,300 g/mol, MFI = 5 g/10 min and viscosity at 40 s^−1^ $\eta_{40s^{-1}}=720\;$Pa·s^−1^) was provided by Flint Hills Resources^®^. Polystyrene (PS) Styron^®^ 615APR (M_w_ = 193,200 g/mol MFI = 14 g/10 min and $\eta_{40s^{-1}}=600$ Pa·s^−1^) was obtained from Americas Styrenics LLC. Styrene-butadiene (SB) diblock and styrene-butadiene-styrene (SBS) triblock copolymers were kindly donated by Kraton^TM^ Corporation, and their specifications are provided in Table 1.

Multi-walled carbon nanotubes (MWCNTs) (Nanocyl^TM^ NC7000) were purchased from Nanocyl S.A. (Sambreville, Belgium). According to the manufacturer, the MWCNTs were produced with the catalytic carbon vapor deposition process, and have an average diameter of 9.5 nm, a length of 1.5 µm, and a surface area of 250–300 m^2^/g.

The studied blend system was PP:PS/70:30 vol.%. A higher content of PP (i.e., 70 vol.%) provided better mechanical properties, while PS is known to generate MWCNT nanocomposites with low percolation threshold and high electrical conductivity [45,46,47]. The polymer blend nanocomposites PP:PS/70:30/MWCNT with and without copolymer were prepared using a Haake Rheomix series 600 OS internal batch mixer (Thermo Scientific Inc., US) connected to a Thermo Scientific Polylab OS platform at 200 °C and 50 rpm using roller blades; 50 rpm corresponds to 40 s^−1^ average shear rate calculated using the approximation of two adjacent sets of concentric cylinders [48]. The volumetric percentage of the blend, the MWCNT, and the copolymer was calculated using the density of each component at the processing temperature of 200 °C and the total volume used in the batch mixer. The blend ratio was kept constant in all the nanocomposites, and the amounts of MWCNT and copolymer were varied. The conditions for mixing were maintained constant for the total 18 min processing time. For mixing the materials, PP and PS were masticated for 3 min, then a mixture of MWCNT and the copolymer, previously dry mixed, was added to the molten PP:PS blend and compounding was continued for an additional 15 min. Thereafter, compression molding was performed using a Carver compression molder (Carver Inc., Wabash, IN) at 200 °C for 15 min under 41 MPa pressure. The samples were molded into a rectangular shape with dimensions 42 × 25 × 0.88 mm^3^.

### 2.3. Materials Characterization

Transmission electron microscopy (TEM) was performed to determine the selective localization of MWCNT in the blend and blend/copolymer systems. 100 nm sections were cut using a Leica EM UC6 ultramicrotome (Leica Biosystems©, Nussloch, Germany) equipped with a diamond knife. The ultramicrotomy was performed at −90 °C in a nitrogen atmosphere. A Tecnai TF20 G2 FEG-TEM (FEI, Hillsboro, OR, USA) electron microscope equipped with a Gatan UltraScan 4000 CCD camera (Gatan, Pleasanton, CA, USA) at 2048 × 2048 pixels was used to image the sections.

Scanning electron microscopy (SEM) was used to study the morphology of the neat PP:PS/70:30, PP:PS/70:30/MWCNT and PP:PS/70:30/MWCNT/copolymer nanocomposites. The samples were etched using tetrahydrofuran (THF) to remove the PS phase. This process was performed for 2 days at ambient temperature. Then, the samples were cryo-fractured in liquid nitrogen and dried in a vacuum oven at 60 °C for 1 day. Before SEM visualization, all samples were coated with platinum in an argon atmosphere to avoid scanning problems, i.e., electrostatic charge accumulation on the surface. The SEM microscope FEI XL30 (FEI, Hillsboro, OR, USA) scan was carried on at an acceleration voltage of 20 kV.

The shear viscosity for the neat type of PP, PS, SB-D1431P, SBS-D1102K, and SB-D0243K systems was measured using a Kayeness (Dynisco) capillary rheometer (Morgantown, PA, USA), Model LCR600 D8052M-115 2046 WVS, in the range of 0–1000 s^−1^. Measurements were taken by a load cell, and the samples were extruded through a 0.508 mm (L/D = 20) die (entrance angle 120°) at 200 °C. The results were analyzed by KARS software, and the Weissenberg–Rabinowitsch correction [49] was performed for each data set.

Electrical resistivity measurements were conducted using two setups according to the resistivity range. For electrical resistivities greater than 10^6^ Ω∙cm, a Keithley 6517 A electrometer connected to a Keithley 8009 test fixture (Keithley Instruments, USA) was used to test the samples. On the other hand, for resistivities lower than 10^6^ Ω∙cm, according to ASTM 257-75 standards, a Loresta GP resistivity meter (MCP-T610 model, Mitsubishi Chemical Co., Tokyo, Japan) connected to an ESP four-pin probe (MCP-TP08P Model, Mitsubishi Chemical Co., Tokyo, Japan) was used. The four-pin probe, which has an inter-pin distance of 5 mm and a pin diameter of 2 mm, was used to minimize the effect of the contact resistance. The applied voltage for all the electrical resistivity measurements was 90 V. Three samples per each composite assay were measured, and the results were averaged.

The rheological behavior was evaluated using a rheometer (MCR 302, Anton Paar). The set-up was equipped with a 25 mm parallel-plate geometry at a gap-size of 0.3 mm. All the oscillatory tests were performed at 200 °C.

## 3. Results and Discussion

### 3.1. Molecular Simulation

**Figure 3:**
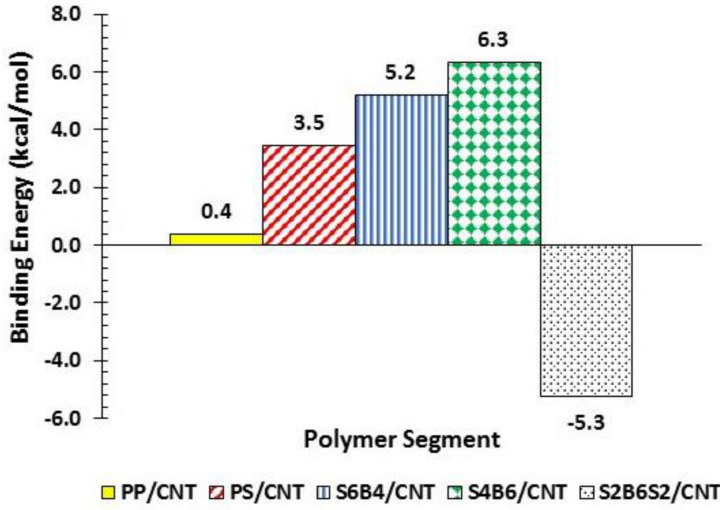
Binding energy of the polymer/CNT and copolymer/CNT assemblies.

A summary of the system energies and orbitals is found in Table S1 and the optimized geometries are shown in Figure S1. Figure 3 shows the binding energy results for polymer/CNT and copolymer/CNT systems. PS had larger binding energy towards CNT than PP; thus, the interaction for PS/CNT was more favorable than for PP/CNT. The favorable interaction of PS/CNT could also be appreciated by the extended configuration of PS (Figure S1b) over the surface of the CNT in comparison to the coiled PP structure when interacting with the CNT (Figure S1a). The diblock copolymers showed the best interaction (larger binding energy) with the CNT in comparison to PS, PP, and the triblock copolymer. The triblock copolymer showed a less favorable interaction with the CNT surface (thermodynamically unstable), as observed in the negative value of the binding energy (see Figure 3). The best interaction was observed for the diblock copolymer with a higher amount of butadiene repeating units (S4B6/CNT).

It was noticed that when the PS segment was connected to the butadiene part of the copolymer, the PS molecules did not take the stiff linear configuration; rather, they took a coiled configuration (Figure S1). Thus, when the copolymer interacted with the CNT (Figure S1c–e), the PS needed more energy to overcome the intramolecular interactions between the blocks in order to interact with the surface of the CNT. On the other hand, since the butadiene remained linear, it could easily access more of the CNT surface than the PS, thus, benefiting the interaction. In a work developed by Luo et al. [50,51] on styrene-butadiene block copolymer rubber (SBR), the molecular simulation showed that increasing the vinyl content in the SSBR diblock enhanced the binding energy towards a graphene nanosheet surface. They also experimentally demonstrated that as the vinyl content increased, the interaction between SSBR and graphene increased, correlating to (1) an increase of glass transition temperature, (2) a decrease of the fractional free volume of the SSBR chains when mixed with the graphene, and (3) a decrease in mobility as proven by dynamic mechanical analysis and positron annihilation lifetime spectroscopy.

### 3.2. MWCNT Localization

**Figure 4:**
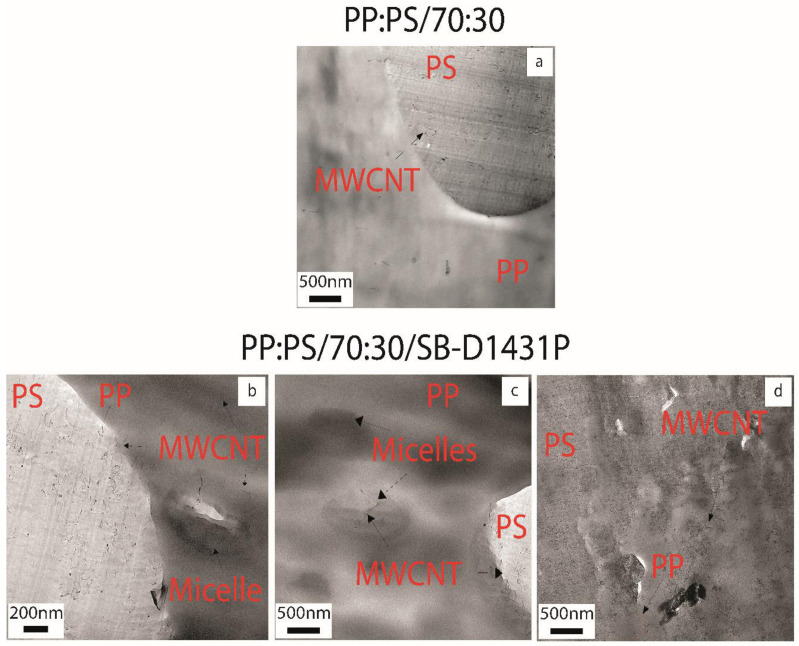

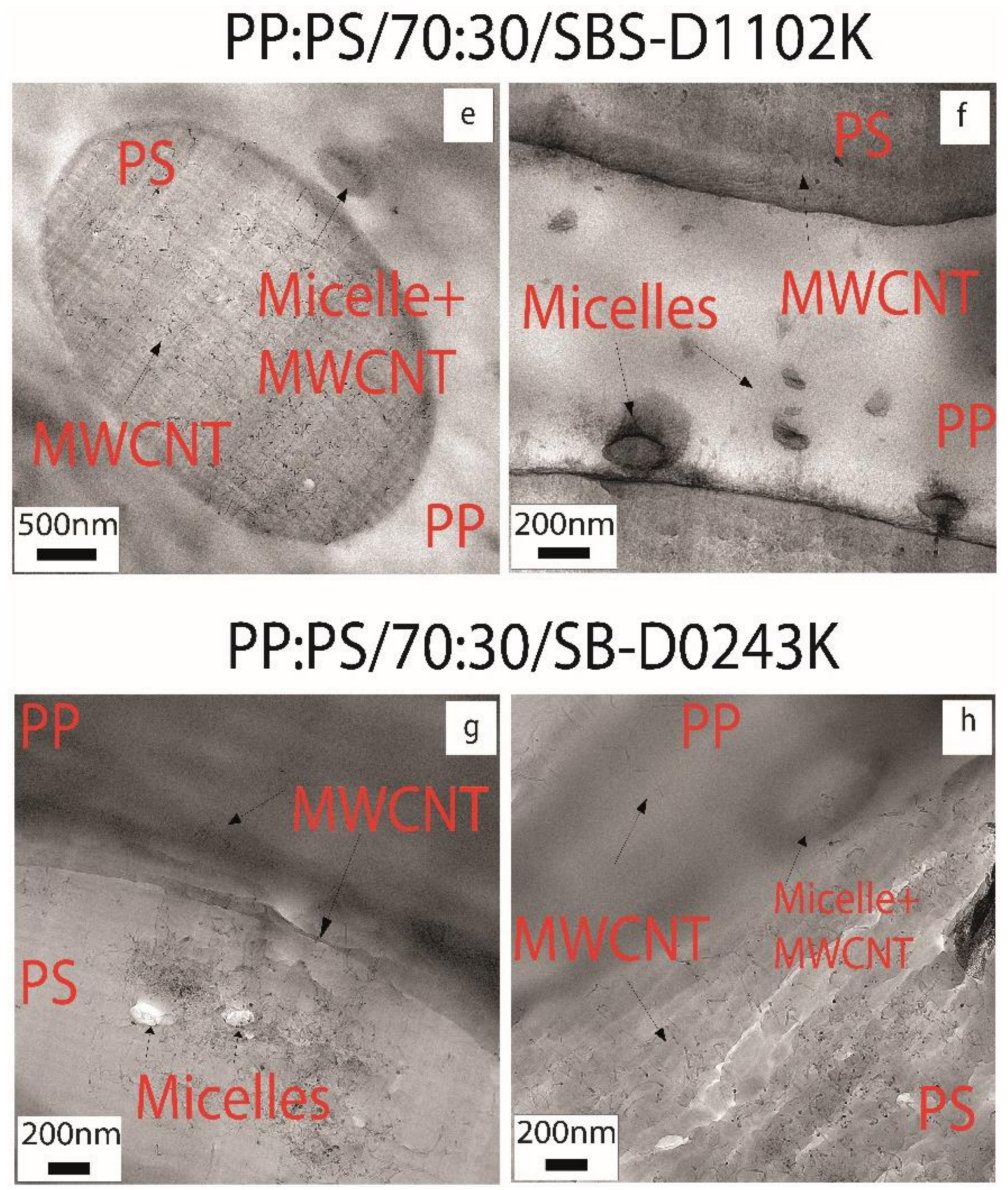
TEM micrographs of 1.0 vol.% multi-walled carbon nanotubes (MWCNT) and 5 vol.% copolymer filled (**a**) PP:PS/70:30, (**b**–**d**) PP:PS/70:30/SB-D1431P, (**e**,**f**) PP:PS/70:30/SBS-D1102K, and (**g**,**h**) PP:PS/70:30/SB-D0243K nanocomposites.

The localization of the MWCNTs in the immiscible blend systems was directly determined using TEM. Figure 4 shows the TEM micrographs of PP:PS/70:30 and PP:PS/70:30/copolymer blend nanocomposites containing 1.0 vol.% MWCNT. The PP and PS domains can be observed in all the micrographs. To investigate the selective localization of the MWCNTs in the blend nanocomposites, the selected area diffraction (SAED) technique was used (see Figure S2). The phase with MWCNTs showed diffuse rings, a characteristic of the amorphous PS phase, while MWCNT-free phase presented diffraction patterns with more defined rings, a feature of PP mixed with copolymers [52].

The TEM image of the blend without copolymer in Figure 4a clearly shows that MWCNTs were mainly within the PS phase, with no MWCNT in the PP phase and only a few MWCNTs were observed at the interphase. This is in line with the simulation results, where it was found that PS had a higher binding energy towards CNT than PP. However, Figure 4b–d shows that most of the MWCNTs were localized in the PS phase upon the addition of block copolymers. However, some MWCNTs were also observed at the interface, inside the micelles, and in the PP phase. It was also noted that some areas in PP did not show the presence of MWCNT, while other areas seemed to have a higher amount of MWCNT, as observed in Figure 4d, where MWCNTs were distributed in PS and PP. When copolymers were mixed with immiscible blends, they had a high tendency to form micelles rather than being adsorbed at the interface [53]. The copolymer could be assembled at the interface by extraction of individual copolymer chains from the micelles or by the demolition of the micelles. Both mechanisms provided free chains to travel to the interface [53,54]. As per the simulation results, diblock copolymers had a high strength of interaction towards the CNT in comparison to the triblock copolymer, PP, and PS. Thus, it was expected for MWCNTs to be localized in the micelles and the interface if interfacial copolymer adsorption was favored.

**Figure 5:**
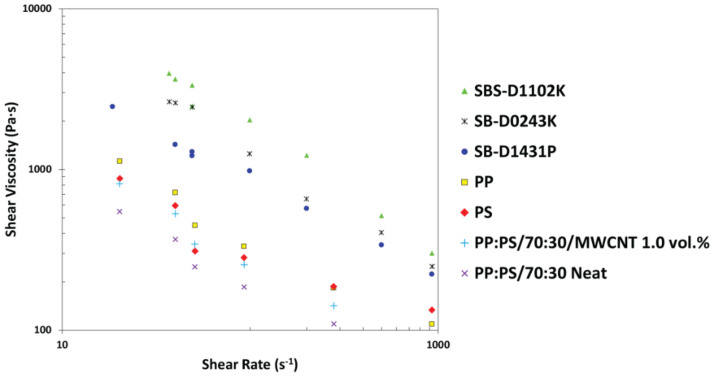
Shear viscosity measured by capillary rheology as a function of shear rate for neat PP, neat PS, and neat copolymers.

The discrepancy between the TEM results and the simulation results might be attributed to the differences in viscosity between the copolymers, PP, and PS. The PS phase had the lowest viscosity among the polymers used (Values from Figure 5: $\eta_{PP}=717\;$Pa∙s, $\eta_{PS}=597$ Pa∙s,$\;\eta_{SB\;D1431P}=1425$ Pa∙s, $\eta_{SBS\;D1102K}=3636$ Pa∙s, and $\eta_{D0243K}=2600$ Pa∙s—the viscosities are referenced to the average shear rate of mixing of 40 s^−1^). The low viscosity of the PS might have facilitated the wetting of MWCNTs, thus decreasing the probability of interaction between MWCNTs and the copolymers, as well as with PP. In addition, it is important to consider that the formation of micelles or the possible assembly of copolymers at the interface might have altered the localization of MWCNTs in the blend. Thus, the mutual interactions between the copolymers, PP, PS, and CNT should also be considered. We used the Hildebrand solubility parameters (δ) (Table 2) for PP, PS, polybutadiene (PBD), and CNT to analyze the quality of interactions between the copolymer, polymers, and MWCNT. The solubility parameters were obtained from the literature [55,56,57,58,59].

Comparing the solubility values in Table 2, the styrene blocks of the copolymer and the PS had a better affinity towards the CNT compared to PP and butadiene block. The styrene and butadiene blocks of the copolymer preferred to interact with the PS and PP phases forming micelles or traveling to the interface, rather than with CNT, given the difference in solubility parameters (i.e., $\left|\delta_{PBD}-\delta_{pp}\right|{\;}^{2}=0.09\;\mathrm{vs}.\;\left|\delta_{PBD}-\delta_{CNT}\right|{\;}^{2}=6.76\;c$al^1^ cm^−3^). Hence, it can be inferred that during mixing, the copolymer chains preferred to interact with PP or PS than remaining with the CNT. Hence, according to the simulation results, CNTs not covered by copolymer chains can migrate to PS phase, which is energetically more favorable than PP. In fact, according to the solubility parameter data, CNTs showed a higher affinity to the styrene block and PS phase; thus, they can preferentially localize in PS and the styrene block of the copolymer and form micelles. TEM results (Figure 4) supported this explanation, since most of the MWCNTs were localized in PS. On the other hand, the presence of some MWCNTs in PP might be due to the migration of MWCNT, arising from micelle formation and copolymer migration to the interface.

### 3.3. Morphology Observation

**Figure 6:**
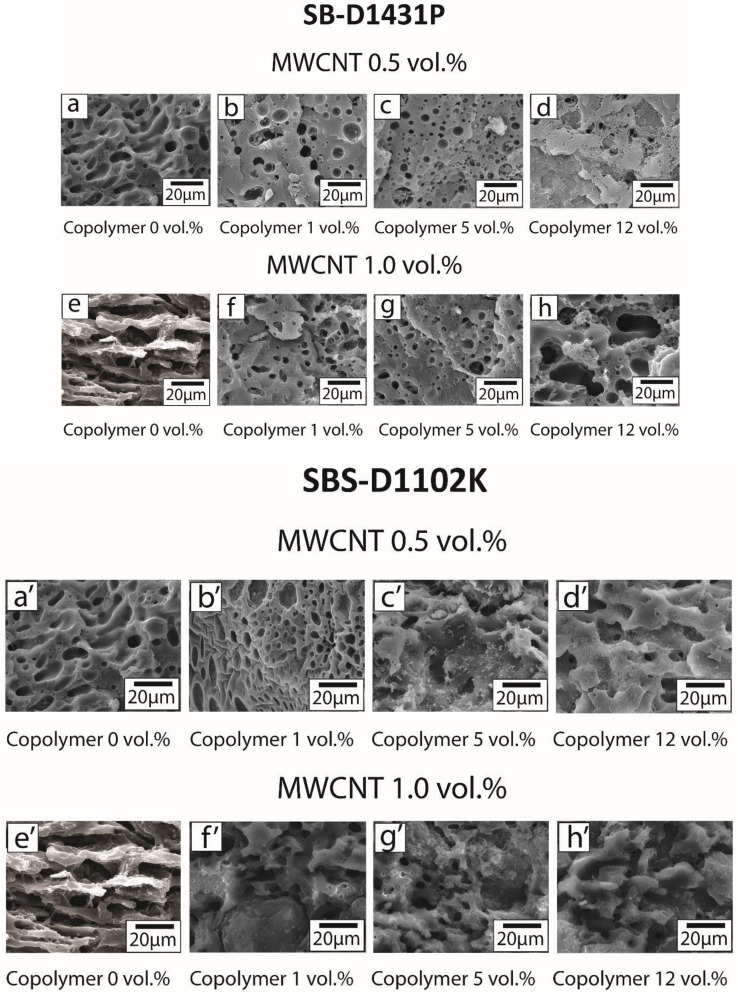

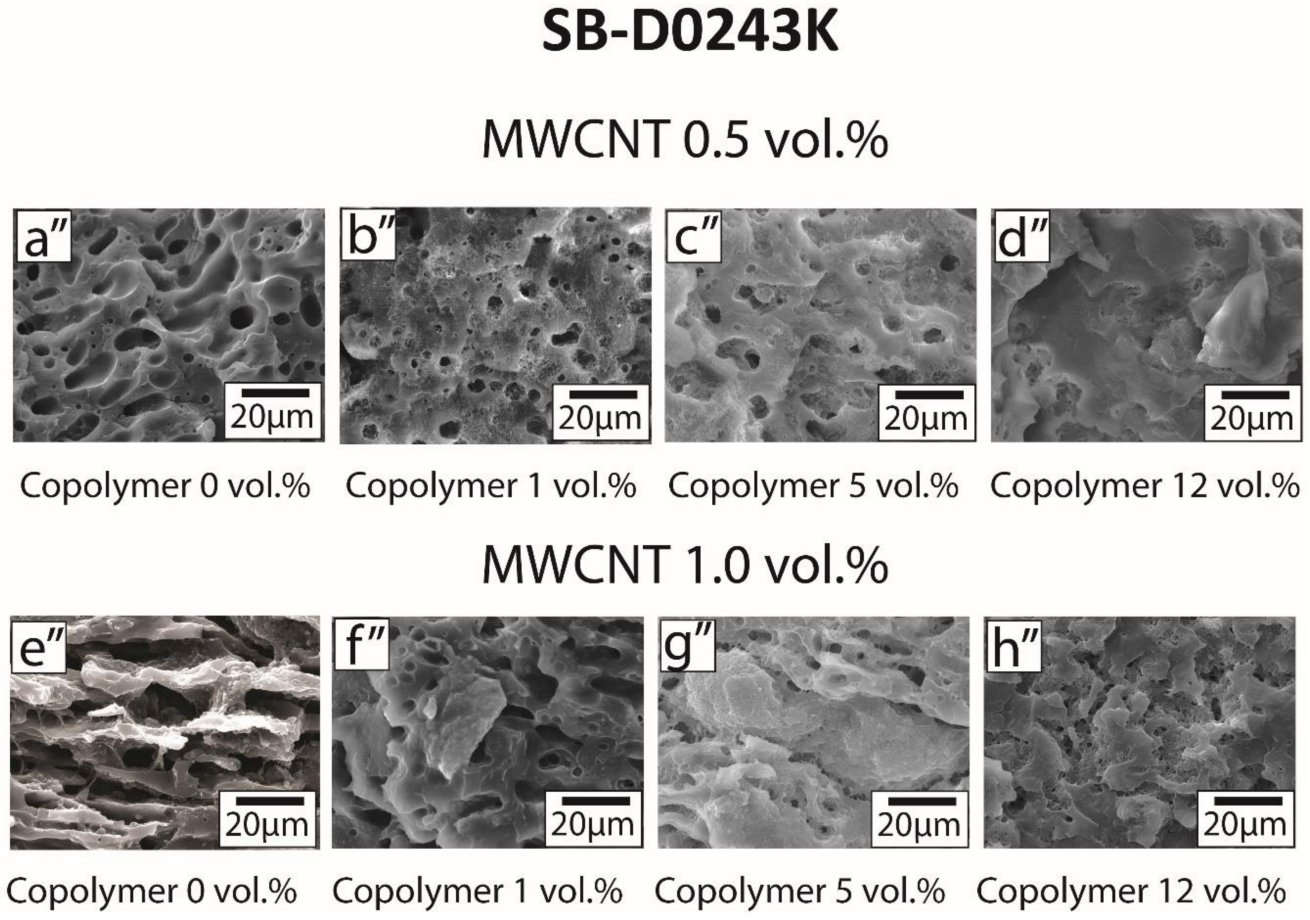
SEM micrographs of (**a**–**d**) PP:PS/70:30/SB-D1431P/MWCNT 0.5 vol.% and (**e**–**h**) PP:PS/70:30/SB-D1431P/MWCNT 1.0 vol.%. (**a’**–**d’**) PP:PS/70:30/SBS-D1102K/MWCNT 0.5 vol.% and (**e’**–**h’**) PP:PS/70:30/SBS-D1102K/MWCNT 1.0 vol.%. (**a”**–**d”**) PP:PS/70:30/SB-D0243K/MWCNT 0.5 vol.% and (**e”**–**h”**) PP:PS/70:30/SB-D0243K/MWCNT 1.0 vol.%.

To determine the morphology changes induced by the addition of block copolymers to the PP:PS/70:30/MWCNT blends, SEM was performed on THF-etched samples. As mentioned in the experimental part, THF removes the PS phase. Figure 6 shows the SEM micrographs of the PP:PS/70:30 blends with the different copolymers. Both PP:PS/70:30/MWCNT 0.5 vol.% and PP:PS/70:30/MWCNT 1.0 vol.% blends (Figure 6a,e—same micrographs as for Figure 6a’,e’, and Figure 6a”,e”) in the absence of block copolymers displayed a deformed and non-spherical PS phase.

In a previous work by our group [24], we showed the ability of MWCNTs at high concentrations to transform the phase morphology from dispersed to co-continuous when MWCNTs were located in PS phase. During the morphological transition from dispersed to co-continuous morphology, the PS/MWCNT domains became elongated and deformed. The selective localization of MWCNTs in the PS phase modified the rheology (e.g., elasticity and viscosity) and, thus, retarded the relaxation dynamics of the PS phase. This deceased the breakup mechanisms. The elongated domains favored the PS/MWCNT percolation in PP. In addition, MWCNTs did not act as barriers during the coalescence processes; instead, they acted as bridges between the droplets. This facilitated the transfer of PS chains from one domain to another, and thus enhanced the droplet coalescence.

Addition of 1.0 vol.% of any of the block copolymers into the PP:PS/70:30/MWCNT 0.5 vol.% (Figure 6b,b’,b”) promoted the formation of smaller and more spherical PS domains. This result showed that the addition of block copolymer effectively suppressed the coalescence by steric stabilization of the interphase [5,39] and/or due to the Marangoni stresses [39,40,41]. The localization of the styrene segment of the block copolymer inside the PS droplet and the butadiene segment in the PP matrix formed a copolymer barrier layer that blocked droplet coalescence and, thus, PS droplets could recoil [5]. For droplet coalescence to occur in the systems, the copolymer had to move out of the interphase [5,60]. Since the viscosity of the block copolymers was2–6 times higher than the PP and PS components (Figure 5), the mobility of the copolymer chains was much less than the PP and PS polymers, retarding the coalescence processes.

For the system with higher MWCNT content, i.e., PP:PS/70:30/MWCNT 1.0 vol.%, addition of 1.0 vol.% of the block copolymers decreased the droplet size, as observed in Figure 6f,f’,f”, but the droplet shape was more irregular. This shows the competition between the effect of MWCNT on modifying the rheology and producing a deformed PS phase, and the effect of block copolymers on preventing PS droplets from coalescing. The deformed shape of the PS droplets upon the addition of MWCNT could also be attributed to the migration of these nanofillers. As MWCNTs migrated from one phase to the other, they pierced the polymer interface, causing interfacial deformations [61]. In addition, PS/MWCNT domains were also deformed during mixing, since the addition of MWCNT increased the viscosity of the PS phase, thereby taking a longer time for the PS/MWCNT to fully recover to a spherical shape. In other words, the existence of nanotubes increased the relaxation time of a deformed droplet to a symmetrical sphere [24].

The addition of copolymers has been shown to undermine the coalescence processes due to the decrease of the interfacial mobility [37,62]. On the other hand, upon addition of block copolymer, the PS domain size decreased in both blends with 0.5 vol.% and 1.0 vol.% MWCNT, confirming that the copolymers were able to migrate from the micelles to the interface between PP and PS. This finding supports the analysis in Section 3.2 about the localization of most of MWCNTs in the PS phase. The copolymer preferred to form micelles or migrate to the interface, as it was energetically more favorable; hence, MWCNTs were free to migrate to the PS phase.

**Figure 7:**
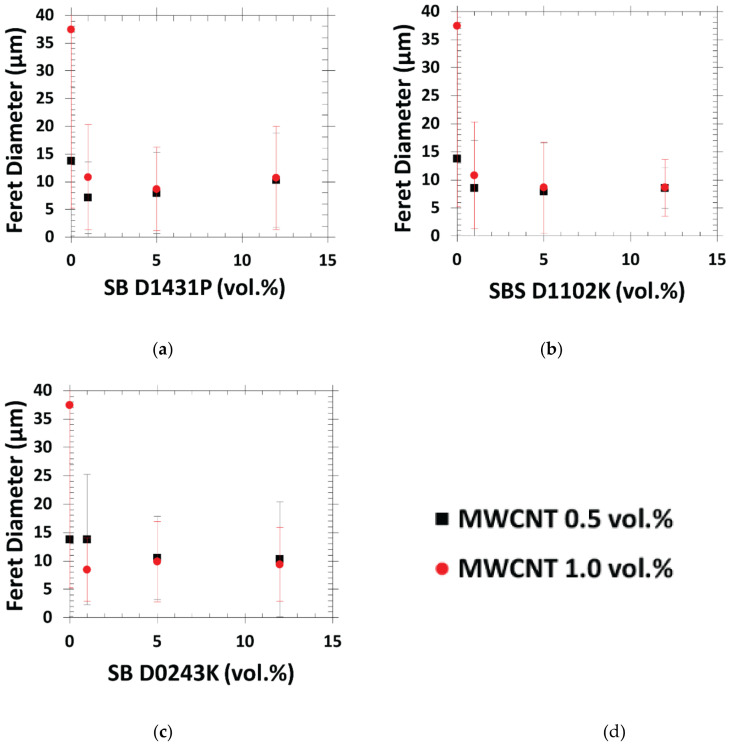
Emulsification curves of 0.5 vol.% and 1.0 vol.% MWCNT filled (**a**) PP:PS/70:30/SB-D1431P, (**b**) PP:PS/70:30/SBS-D1102K, and (**c**) PP:PS/70:30/SB-D0243K. (**d**) Description of the MWCNT concentration. The upper and lower horizontal axes in each plot corresponded to the copolymer concentration in the minor phase (PS) and to the copolymer overall concentration in the nanocomposite, respectively.

**Figure 8:**
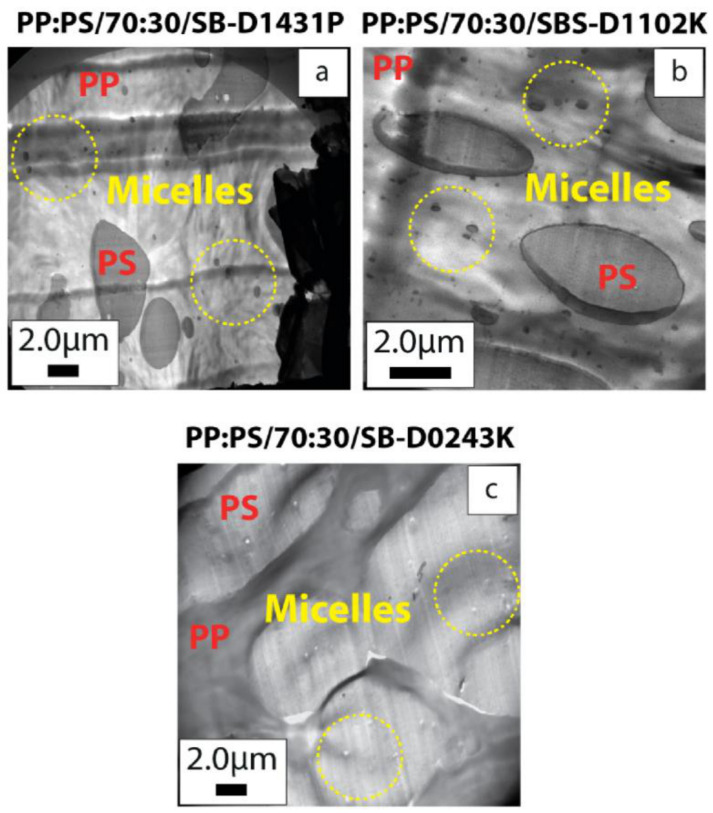
TEM micrographs showing micelles formation in 1.0 vol.% MWCNT and 5 vol.% copolymer filled (**a**) PP:PS/70:30/SB-D1431P, (**b**) PP:PS/70:30/SBS-D1102K, and (**c**) PP:PS/70:30/SB D0243K.

The differences between the three block copolymers in terms of the PS/MWCNT droplet size were quantified in the emulsification curves, displayed in Figure 7, where both MWCNT 0.5 vol.% and 1.0 vol.% were considered. The emulsification curve was developed for the emulsions; however, Favis et al. [63,64] showed it to be useful for the polymer blends. For all the three systems, the critical concentration at which the droplet size had the steepest reduction corresponded to 1.0 vol.% of the block copolymer. At higher copolymer concentrations, the PS droplet size did not change significantly, indicating copolymer saturation at the interphase. As a result, at high copolymer concentrations, copolymer micelles formed in the polymeric phases, as shown in Figure 8. Furthermore, diffusion times in polymers were much longer than Newtonian fluids; thus, the migration of the copolymers to the interphase was sluggish, and thermodynamically they preferred to remain as micelles.

Furthermore, from the emulsification curve in Figure 7, we can observe that increasing the MWCNT content from 0.5 vol.% to 1.0 vol.% did not affect the compatibilization effect of the copolymers. Although the rheology of the PS phase was influenced more when MWCNT concentration was increased, the interactions of the copolymer block segments with PP and PS phases dominated the morphology development in the PP:PS/70:30/MWCNT/copolymer systems; thus, a decrease in domain size was observed (see Figure 6). Figure S3 shows additional SEM micrographs of PP:PS/70:30/Copolymer/MWCNT at a constant 1.0 vol.% block copolymer content (i.e., at the critical concentration in the emulsification curve) at different MWCNT concentrations. Increasing MWCNT did generate significant morphological changes for the PP:PS/70:30/copolymer/MWCNT blends.

It was also observed that in the blend systems PP:PS/70:30/SB-D1431P (Figure 8a) and PP:PS/70:30/SB D1102k (Figure 8b), the micelles were mainly localized in the PP phase, while in the system PP:PS/70:30/SB-D0243K (Figure 8c), the micelles were mainly located in the PS phase. The selective micelle formation in PP or PS was driven by the minimization of the interfacial energy between the copolymer segments and the homopolymer in contact with the copolymer [65]. Adedeji et al. [66] found that the aggregation of poly(styrene-b-poly(methyl methacrylate)) (PS-b-PMMA) block copolymers in PMMA/poly(cyclohexyl methacrylate) (PCHMA) depends on the molecular weight of the droplets. They observed that the block copolymers started to form micelles before they saturated the interfaces. Micelles occurred in the poly(methyl methacrylate) phase when its molecular weight was lower than the PMMA block. Hlavata et al. [67] studied the effect of styrene block length of styrene-butadiene copolymers in the compatibilization of PP:PS blends. They found that the most important factor controlling the localization of the SB copolymers at the interface is the length of the styrene blocks and not the number of blocks (diblock, triblock, etc.). If the molar mass of the styrene block was more than the molar mass needed to form entanglements with the PS phase, then the copolymer would be mainly localized in the PS. On the other hand, if the styrene block length was less than the molar mass needed to form entanglements with the PS, then the copolymer will localize at the interface resulting in a more effective compatibilization. On the other hand, molecular simulation results in Section 3.1 showed that the diblock segment with a higher content of butadiene (S4B6), which corresponds to SB D0243K, had the highest binding energy towards the CNT surface in comparison to the other copolymers. This might change the micelle equilibrium assembly in the PP:PS/70:30 blend and make micelle localization in the PS phase when SB D0243K copolymer was used.

### 3.4. Electrical Properties

**Figure 9:**
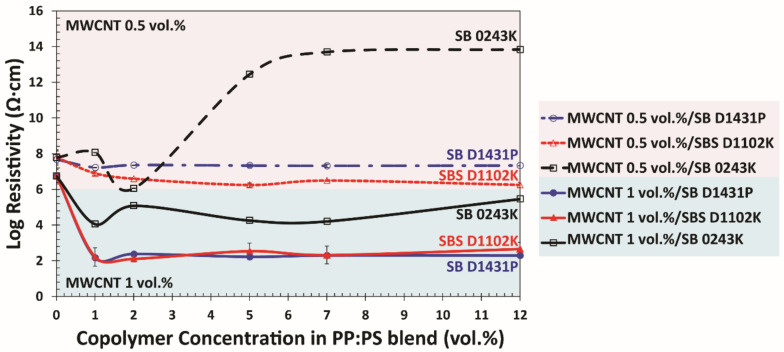
Electrical resistivity of blend nanocomposites with 0.5 vol.% (pink-shaded area) and 1.0 vol.% (blue-shaded area) MWCNT concentration as a function of copolymer content.

The electrical resistivity (ρ) is an indication of the material’s resistance to conduct electrical current [66]. ρ of polymer nanocomposites follows a percolation-like behavior, and a critical value of filler loading must be incorporated to have a network of conductive fillers formed. This critical concentration is known as percolation threshold. The balance between nanofiller–nanofiller and filler–polymer interactions is critical for the formation of networks inside the blend [68,69,70,71]. Considering that the addition of any of the block copolymers decreased the PS domain size and thus deteriorated the interconnectivity among PS domains (Section 3.3), it was expected that the addition of block copolymers would cause an increase in electrical resistivity (ρ), due to the disruption of the PS/MWCNT percolative network inside the PP phase. Nevertheless, we interestingly found that the addition of diblock SB-D1431P and the triblock SBS-D1102K to the PP:PS/70:30/MWCNT 0.5 vol.% and PP:PS/70:30/MWCNT 1.0 vol.% led to a significant decrease in ρ (Figure 9).

**Figure 10:**
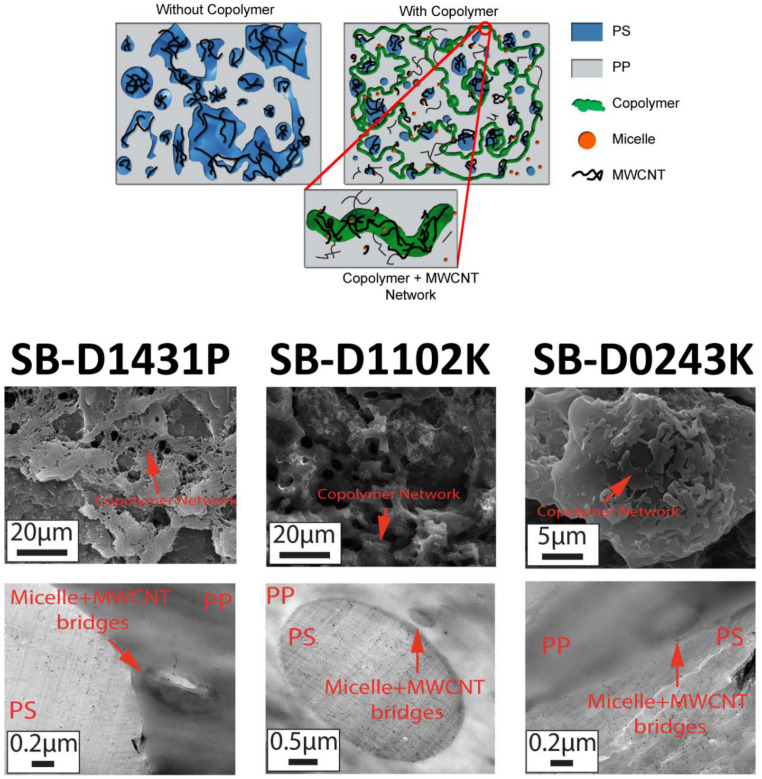
Schematic of the conductive network in PP:PS/70:30/MWCNT nanocomposites without and with block copolymers. SEM micrographs show the copolymer network and TEM micrographs display that MWCNTs bridge the micelles and PS phase.

It is proposed that the improvement in the electrical conduction of the blend nanocomposites upon the addition of the block copolymers might have been due to the interconnected assembly of the PS droplets, copolymers, PP, and micelles containing MWCNT (see schematic in Figure 10). In fact, the combination of percolated PS droplets, micelles, PP, and copolymer containing MWCNT formed a double percolated structure capable of electron transport. In Section 3.2, we observed that the blend containing SB-D1431P copolymer showed some areas where PP and PS phases are interconnected by MWCNTs (Figure 4d). In addition, some MWCNTs were observed bridging micelles and PS phase (Figure 4b,h and Figure 10). The copolymers could also form the third phase at a critical concentration.

Macaúbas and Demarquette [35] showed that when a triblock SBS was added into a PP:PS blend at a concentration of 15 wt.% relative to the dispersed phase (PS), SBS segregated and formed the third phase. Although from TEM micrographs, the third phase of copolymer could not be distinguished, SEM micrographs (Figure 10) showed that the copolymers might have formed a network in the blend. In addition, since MWCNTs were observed inside micelles and at the interface, it is possible to infer that MWCNTs could also be present in the copolymer. The network can be appreciated in Figure 10, where some regions of the samples showed an interconnected phase among PS domains (etched phase), which also extended into the PP phase.

Furthermore, it should be noted that for the blends containing diblock SB-D1431P and triblock SBS-D1102K, the steepest decrease in resistivity coincided with the critical copolymer concentration at which the PS droplet size significantly decreased (Figure 7). Unchanged resistivity at copolymer contents higher than the critical concentration also correlated to the insignificant changes in morphology at higher copolymer contents. However, for the blend with diblock SB-D0243K, there was an anomalous increase in resistivity at 0.5 vol.% MWCNT that occurred at a higher copolymer content (2.0 vol.%) than the critical concentration (1.0 vol.%). This was due to the greater number of micelles inside the PS phase in the PP:PS/70:30/MWCNT/SB-D0243K system (Figure 8c); these micelles could not contribute to the formation of interconnected networks among the PS/MWCNT domains inside the PP phase. On the other hand, we also observed that in all the copolymer/blend systems with a copolymer concentration of 1.0 vol.% (critical concentration), increasing the amount of MWCNT did not result in significant changes in the electrical behavior of the PP:PS/70:30/copolymer/MWCNT nanocomposite compared to the PP:PS/70:30/MWCNT nanocomposite (Figure S4). This result matched with the minor morphological changes that occurred in the PP:PS/70:30/MWCNT nanocomposite upon increasing the MWCNT content (Figure S3).

### 3.5. Rheology

In this section, nonlinear rheological tests were used to further characterize the morphological features of the polymer blend nanocomposites without and with copolymers [72,73,74]. Strain sweep measurements were used to investigate both inter-cycle (changes between successive oscillations) and intra-cycle (changes in each cycle of oscillation) viscoelastic behavior of the samples.

**Figure 11:**
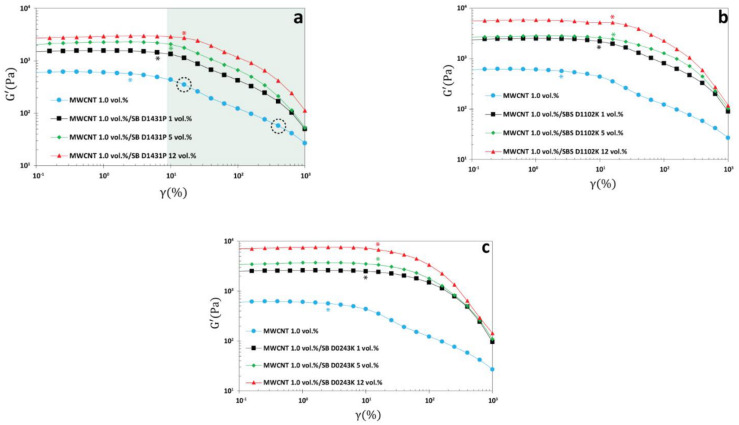
Oscillatory amplitude sweep results of PP:PS/70:30/MWCNT 1.0 vol.% and PP:PS/70:30/MWCNT 1.0 vol.%/copolymer at different block copolymer concentrations. (**a**) PP:PS/70:30/MWCNT 1 vol.%/SB D01431P, (**b**) PP:PS/70:30/MWCNT 1 vol.%/SBS D1102K, and (**c**) PP:PS/70:30/MWCNT 1 vol.%/SB D0243K. The star symbols correspond to the transition from linear to nonlinear regimes. Shaded area in (**a**) highlight the two-step-yielding. Dashed circles represent the two yielding points in the PP:PS/70:30/MWCNT 1.0 vol.% in the absence of copolymer. Strain sweep tests were performed at 200 °C and an angular frequency of ω = 1.0 rad/s.

Based on Figure 11, it can be appreciated that the addition of 1.0 vol.% of any of the block copolymers into the PP:PS/70:30/1.0 vol.% MWCNT led to a significant increase in the value of the plateau-storage modulus (G′) in the linear viscoelastic limit. The enhancement of G′ could be attributed to the increase in the interfacial area and elasticity [72,73] deriving from the morphological evolution given by the decrease of the domain size when the copolymers are added. Additionally, the stitching effect of the block copolymer at the PP:PS interphase provided a better stress transfer through the interface, thus impacting the storage modulus positively.

The other noticeable viscoelastic change by the addition of the copolymers was the location of the critical strain amplitude$\;\gamma_{0}^{*}$ (i.e., transition from linear regime to nonlinear regime shown by star symbols in Figure 11). Interestingly,$\;\;\gamma_{0}^{*}$ shifted to higher strain amplitudes when the copolymers were added to the blend nanocomposites. Under sufficiently large strain amplitudes, the droplets were deformed and elongated; hence, breakup and coalescence could happen. This implies that the anchored blocks of the copolymer at the interphase were also stretched or unraveled from each of the phases [72] resulting in an increase in the resistance of the nanocomposite to flow (i.e., increase in the value of the onset of the linear to non-linear transition).

Moreover, the polymer blends with and without copolymers followed different inter-cycle nonlinear scenarios in sufficiently large deformations. That is, the blend samples without the copolymers followed a weak two-step yielding (i.e., drop–plateau–drop in the value of G′) process upon exceeding the limit of linearity (shaded region in Figure 11a). While the observed two-step yielding switched to one step yielding in the presence of the copolymers. Figure S5 verifies that the two-step yielding stems from the nature of each phase as the pure PP:PS/70:30 blend without MWCNTs also featured two-step yielding. Moreover, as it can be seen in Figure S5, the addition of MWCNTs sharpened the two-step yielding process. Hence, the stitching effect of the copolymers led to a more uniform deformation in the bulk of the samples, weakening the two-step yielding process. Hence, based on Figure 11, addition of copolymers significantly changed the value of the linear viscoelastic parameters, the onset of the nonlinearity, and the type of the inter-cycle nonlinear behavior.

In our previous works we showed the sensitivity of intra-cycle nonlinear viscoelastic response to subtle changes in the microstructure of different systems [74,75,76,77,78,79,80]. Hence, more information regarding the morphological changes of polymer blends with and without copolymers based on intra-cycle nonlinear viscoelasticity is provided in this section. To this aim, we used the stress decomposition method [81] to analyze the output stress waveform and to deliver physical interpretation based on the nonlinear viscoelastic behavior. In the stress decomposition method, the total shear stress is decomposed into elastic $(\tau^{\prime }$) and viscous ($\tau^{\prime \prime })$. Thus, the total shear stress can be expressed as:

(2) $$\sigma \left(t\right)=\tau^{\prime }\left(t\right)+\tau^{\prime \prime }\left(t\right),$$

where the elastic stress component ($\tau^{\prime }$) and viscous stress component ($\tau^{\prime \prime }$) are an odd function of normalized strain ($x\left(t\right)=\;\frac{\gamma \left(t\right)}{\gamma_{0}}$) and normalized strain rate ($y\left(t\right)=\;\frac{\dot{\gamma }\left(t\right)}{{\dot{\gamma }}_{0}}$), respectively. Lissajous–Bowditch loops, which demonstrate the output stress waveform against strain or strain-rate, could be co-plotted by elastic stress component ($\tau^{\prime }$) and viscous stress component ($\tau^{\prime \prime }$). Lissajous–Bowditch loops provide us with qualitative interpretation of the viscoelastic properties. For instance, the emergence of any distortion in the ellipsoidal shape of the Lissajous–Bowditch loops marks the occurrence of nonlinearity in the examined system. In the following section, we distinct our samples based on the Lissajous–Bowditch loops. We also provide more quantitative information about the nonlinearity of the polymer blend nanocomposite.

**Figure 12:**
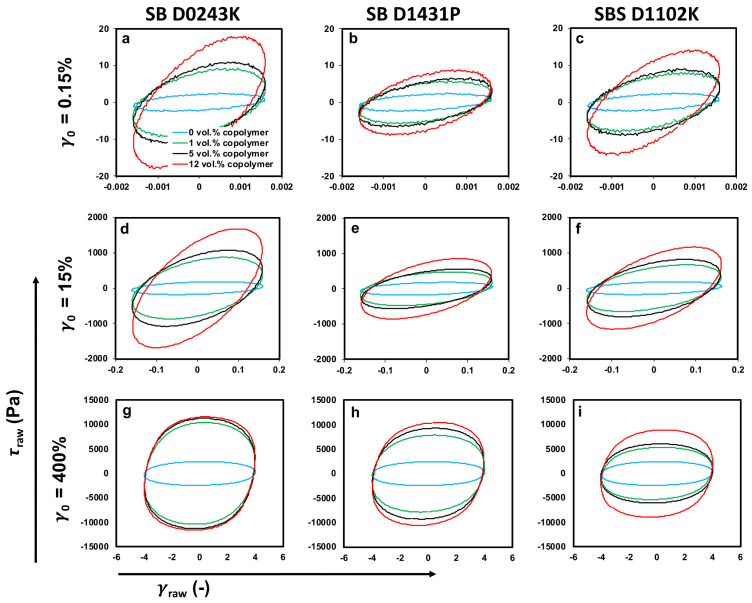
(**a**–**i**) Elastic Lissajous–Bowditch loops for PP:PS/70:30/MWCNT 1 vol.% and PP:PS/70:30/MWCNT 1 vol.%/copolymers. Projections on the elastic (τ − γ) plane are presented at strain amplitudes of $\gamma_{0}\;=$ 0.15%, 15%, and 400% and an angular frequency of ω = 1 rad/s at 200 °C.

**Figure 13:**
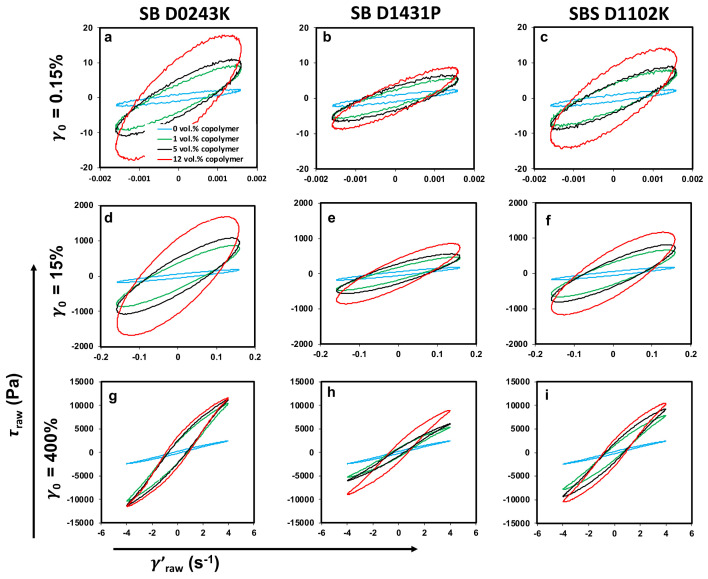
(**a**–**i**) Viscous Lissajous–Bowditch loops for PP:PS/70:30/MWCNT 1 vol.% and PP:PS/70:30/MWCNT 1 vol.%/copolymers. Projections on the viscous (τ − $\frac{d\gamma }{dt}$) plane are presented at strain amplitudes of $\gamma_{0}=$ 0.15%, 15%, and 400% and an angular frequency of ω= 1 rad/s at 200 °C.

Lissajous–Bowditch loop (simplified as Lissajous loops) projections on the elastic (τ-γ) and viscous (τ-$\frac{d\gamma }{dt}$) planes at strain amplitudes of $\gamma_{0}=$0.15%, 15.0%, and 400.0%, and an angular frequency of ω=1.0 rad/s are presented in Figure 12 and Figure 13. As can be seen, samples showed an ellipsoidal shape in the linear regime ($\gamma_{0}=\;$0.15 %) on both elastic and viscous projections, which is a typical response of viscoelastic materials as both input strain/strain-rate and output stress signal could be defined by a simple sinusoidal function by the first harmonic. However, the excitation of higher harmonics in the nonlinear region led to distortion in Lissajous loops.

As can be seen in Figure 12 and Figure 13, Lissajous–Bowditch loops’ shapes were sensitive to any change in the morphology of the polymer blends. By adding the copolymers, the major axis of the ellipses rotated counter-clockwise (signifying enhancement in the value of complex modulus/viscosity at $\gamma_{0}=\;$0.15%), and the area of the Lissajous curves increased. These results reveal that the polymer blend nanocomposites containing copolymers exhibited different intra-cycle viscoelastic behavior at small strain amplitudes, i.e., $\gamma_{0}=\;$0.15%, in the linear viscoelastic region compared to blend nanocomposites without copolymers.

Increasing the strain amplitude beyond the linear viscoelastic framework, $\gamma_{0}=\;$400%, led to a distortion in the ellipsoidal shape of Lissajous curves, signifying the existence of higher harmonics in the output stress waveform. Incorporating copolymers resulted in a more distortion in the Lissajous loops, and the shape of the elastic loops changed into a rectangular shape as the amount of the modifiers increased. This means that samples containing copolymers revealed remarkable nonlinear viscoelasticity in the deep nonlinear region (e.g., strain amplitude of $\gamma_{0}=\;$400%). The same conclusion could be drawn according to the viscous projection of the Lissajous loops, i.e., the distortion is more obvious in the viscous projection in the presence of the copolymers, indicating higher nonlinear behavior.

Moreover, the area of the total stress curve in elastic projection could be used to determine the dissipated energy per unit volume [82]. As can be seen in Figure 12, the area of the Lissajous–Bowditch loops in the elastic projection increased dramatically by adding the copolymers, marking that more energy being dissipated in the copolymer incorporated samples. This phenomenon could be attributed to a change in the viscosity, interfacial tension, and morphology of the blend system in the presence of the copolymers.

**Figure 14:**
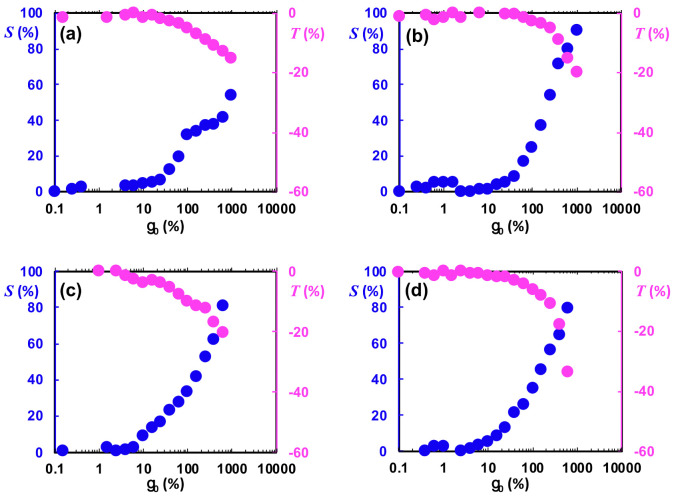
Elastic (*S*) and viscous (*T*) intra-cycle nonlinearity indices as a function of imposed strain amplitude for (**a**) PP:PS/70:30/MWCNT 1 vol.%, (**b**) PP:PS/70:30/MWCNT 1 vol.%/SB D0243K 5 vol.%, (**c**) PP:PS/70:30/MWCNT 1 vol.%/SB D1431P 5 vol.%, and (**d**) PP:PS/70:30/MWCNT 1 vol.%/SBS D1102K 5 vol.% blend nanocomposites.

Figure 14 depicts the intra-cycle strain-stiffening ratio (*S*) and shear thickening ratio (*T*) indices for samples, which is defined as $S\equiv \frac{G_{L}^{\prime }-G_{M}^{\prime }}{G_{L}^{\prime }}$ and$\;T\equiv \frac{\eta_{L}^{\prime }-\eta_{M}^{\prime }}{\eta_{L}^{\prime }}\;$, respectively [83]. $G_{L}\prime$ (large strain modulus) is the secant of the stress at the point where deformation is maximal ($\gamma =\gamma_{0}$) and defined as ${\left.\frac{\tau }{\gamma }\right|}_{\gamma =\pm \gamma_{0}}\equiv G\prime_{L}$, and $G_{M}^{\prime }$ (minimum strain modulus) is the derivative of the stress at the point where deformation takes a zero value (γ=0) and is defined as ${\left.\frac{d\tau }{d\gamma }\right|}_{\gamma =0}\equiv G\prime_{M}$. In the linear regime, where the contribution of the higher harmonics is zero, both $G_{L}^{\prime }$ and $G_{M}^{\prime }$ converge to the linear elastic modulus (G′). Similar to the elastic measures, a set of dynamic viscosities have been defined as minimum rate dynamic viscosity ${\left.\frac{d\tau }{d\dot{\gamma }}\right|}_{\dot{\gamma }=0}\equiv \eta \prime_{M}$ and large rate dynamic viscosity ${\left.\frac{\tau }{\dot{\gamma }}\right|}_{\dot{\gamma }=\pm {\dot{\gamma }}_{0}}\equiv \eta \prime_{L}$, where they converge to the real viscosity (η′) in the linear viscoelastic regime.

As can be seen in Figure 14, both *S* and *T* indices were close to zero in the limit of the linear framework (small strain amplitudes). In the nonlinear regime, a positive *S* index and negative *T* index revealed intra-cycle strain-stiffening and intra-cycle shear-thinning behavior, respectively. The deviation of these indices from zero at higher strain amplitudes showed the extent of the non-linearity. Filipe et al. [84] showed that both diameters of the dispersed phase and the existence of compatibilizer at the interface could affect the nonlinear viscoelastic behavior of the blend systems. The authors also mentioned that the contribution of the second factor is much more significant. A higher value of *T* and *S* indices in the deep nonlinear region for blends containing copolymers compared to polymer blends without copolymer verified their findings (see Figure 14).

## 4. Conclusions

In this study, we investigated the effect of three different block copolymers with various styrene and butadiene contents and structures (diblock and triblock) on the morphological, electrical, and rheological properties of PP:PS/70:30/MWCNT blend nanocomposites. The copolymers effectively decreased the PS domain size in the blend nanocomposites. The copolymers at the interphase provided steric hindrance against the coalescence of PS domains. The increase of MWCNT content induced co-continuity in PP:PS blends; however, this did not affect the migration of the copolymer to the interphase. Molecular simulation and solubility parameter analyses were employed to understand and predict the MWCNT localization in the PP:PS/70:30/copolymer system. The simulation predicted that diblock copolymers had higher binding energy towards CNT surface, in comparison to PP and PS, and indicated that the triblock copolymer/CNT interaction was thermodynamically unfavored. Hildebrand solubility parameters showed that the block copolymer segments preferred to interact with PP or PS rather than with MWCNT. This implies that when copolymers migrate to the interphase, they would expel MWCNTs, allowing them to migrate to the PS phase. However, some MWCNTs were observed in the micelles, the interface, and PP phase. This localization might be attributed to the migration of MWCNTs between the phases due to copolymer assembly in micelles and at the interface.

The rearrangement of MWCNTs in the blend upon addition of the copolymers enhanced the electrical conductivity (decrease in electrical resistivity) of the PP:PS/70:30/copolymer/MWCNT nanocomposite. The integrated network formed by the copolymer, PS droplets, PP, and micelles containing MWCNTs led to a significant decrease of 5 orders of magnitude in electrical resistivity (i.e., increase in electrical conductivity) for the MWCNT concentration of 1.0 vol.%. The significant improvement in electrical conductivity made these nanocomposites promising candidates for applications such as electromagnetic interference shielding and electrically conductive films. Moreover, the rheological results of this work verified that the stress waveform considerably depends on the type of the copolymer, signifying that Lissajous–Bowditch plots can be used as a strong tool to distinguish polymer blends with different morphologies.

## Figures and Tables

**Table 1:** Properties of SB and SBS copolymers.

Polymer	Reference	Manufacturer	Styrene Content wt.%	Shear Viscosity at 200 °C & 40 s^−1^ (Pa∙s)	Specific Gravity
**SBS**	D1102K	Kraton	26.8–30	3640	0.94
**SB**	D0243K	Kraton	31–36	2600	0.94
**SB**	D1431P	Kraton	75	1430	1.01

**Table 2:** Hildebrand solubility parameters of PP, PS, (polybutadiene) PBD, and CNTs. Data were obtained from the literature.

Polymer	Hildebrand Solubility (*δ*)(cal^1/2^ cm^−3/2^)
PP	8.7 [55]
PS	8.9 [55]
PBD	8.4 [55]
SWCNT	Theoretical: Zigzag (6,0) 8.3–Zigzag (15,0) 9.6 [56] Experimental: HiPCO SWCNT 11.4 [57]
DWCNT	Theoretical: 11.1 [56]
MWCNT	Theoretical: 10.5–11.3 [56] Experimental: Arc produced CNT: 10.5–11.2 [58] Experimental: CVD produced CNT (Nanocyl NC7000): 10.3 [59]

## Data Availability

The data presented in this study are available on request from the corresponding author.

## Supplementary Materials

The following are available online at https://www.mdpi.com/2073-4360/13/2/230/s1, Table S1 provides a summary of molecular simulation results. Figure S1 provides information about the molecular simulation optimized geometries and molecular orbitals, respectively. Figure S2 displays the selected area diffraction (SAED) pattern for the blend nanocomposites and compares between PP and PS phases. Figure S3 shows SEM micrographs of PP:PS/70:30 blend at different MWCNT concentrations and 1.0 vol.% copolymer content. Percolation curves at 1.0 vol.% copolymer concentration are displayed in Figure S4. Figure S5 shows the strain sweep result of pure polymer blend with and without MWCNT.
