# Supplementary material for: Morphology Evolution, Molecular Simulation, Electrical Properties, and Rheology of Carbon Nanotube/Polypropylene/Polystyrene Blend Nanocomposites: Effect of Molecular Interaction between Styrene-Butadiene Block Copolymer and Carbon Nanotube
Source: Polymers (Basel). 2021 Jan 11;13(2):230. doi: 10.3390/polym13020230 (PMC7827940; doi:10.3390/polym13020230)
Supplement: Supplementary file 1 [file polymers-13-00230-s001.pdf]

Morphology Evolution, Molecular Simulation, Electrical Properties, and Rheology of Carbon Nanotube/Polypropylene/Polystyrene Blend Nanocomposites: Effect of Molecular Interaction between Styrene-Butadiene Block Copolymer and Carbon Nanotube

Ivonne Otero Navas[1], Milad Kamkar[1,2], Mohammad Arjmand[2] and Uttandaraman Sundararaj[1*]

[1] Department of Chemical and Petroleum Engineering, Schulich School of Engineering, University of Calgary, Calgary, Alberta, Canada
[2] School of Engineering, University of British Columbia, Kelowna, BC, Canada V1V 1V7
* Correspondence: Author: u.sundararaj@ucalgary.ca (Uttandaraman Sundararaj)

1. Molecular Simulation

1.1 Summary of Simulation Results

Table S1. Summary of the ground state energy, energetic values of HOMO (Highest Occupied Molecular Orbital) and LUMO (Lowest Unoccupied Molecular Orbital), binding energy and number of imaginary frequencies for the studied systems.

System	Energy (Ha)	HOMO (eV)	LUMO (eV)	Band Gap (eV)	Binding Energy (kcal/mol)	Number of Imaginary Frequencies
**PP**	-0.25577	-10.09	3.90	14.00	-	0
**PS**	0.25947	-9.04	0.52	9.55	-	0
**S6B4**	0.18333	-9.16	0.30	9.46	-	0
**S4B6**	0.13919	-9.17	0.38	9.54	-	0
**S2B6S2**	0.13697	-9.25	0.38	9.63	-	0
**CNT**	4.44422	-5.82	-4.90	0.92	-	0
**PP/CNT**	4.18785	-5.82	-4.89	0.93	0.4	0
**PS/CNT**	4.69818	-5.82	-4.88	0.94	3.5	0
**S6B4/CNT**	4.61928	-5.84	-4.90	0.94	5.2	0
**S4B6/CNT**	4.57332	-5.83	-4.89	0.94	6.3	0
**S2B6S2/CNT**	4.58957	-5.76	-4.97	0.79	-5.3	0

1.2 Ground State Geometries of the Studied Systems

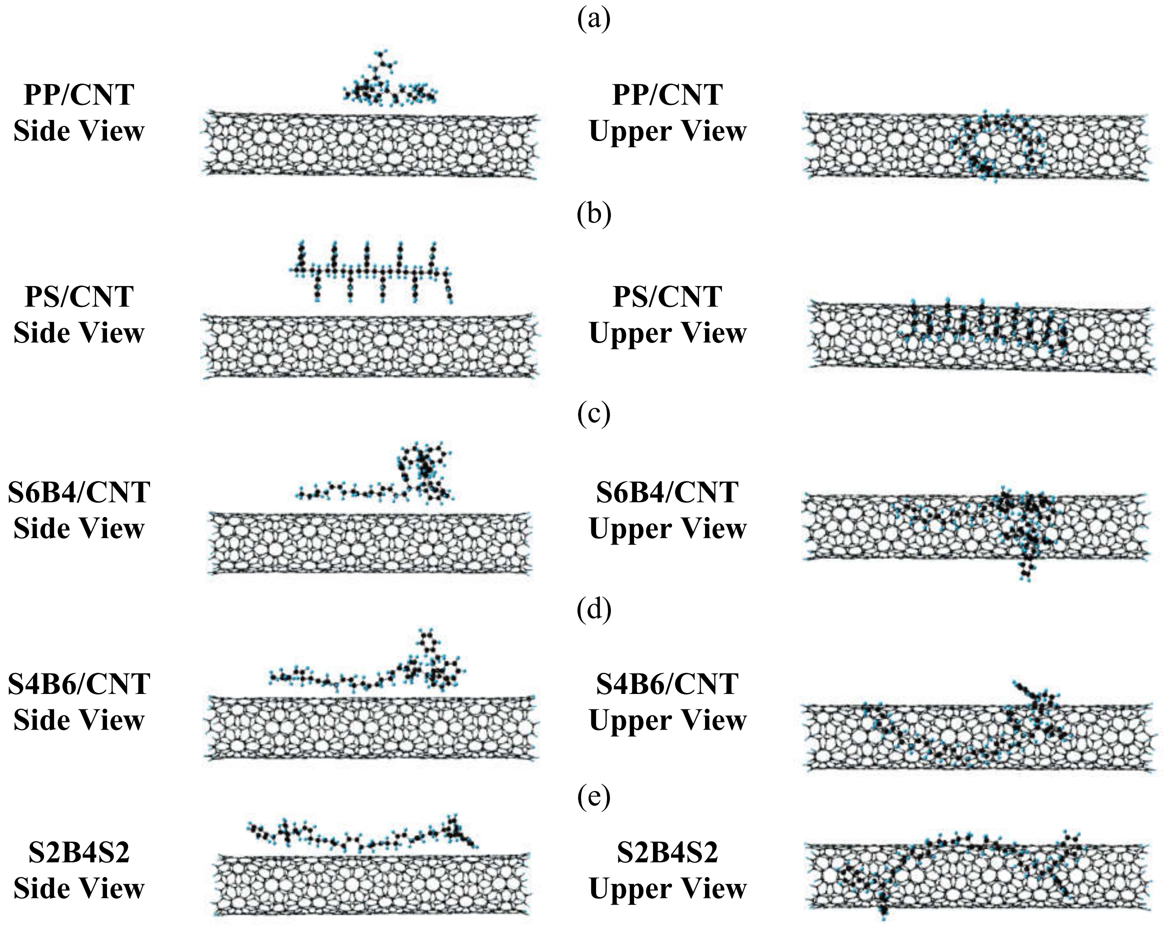

Figure S1. Ground state geometries of PP, PS, armchair-CNT, zigzag-CNT, PP/CNT and PS/CNT systems.

2. Selected area electron diffraction (SAED)

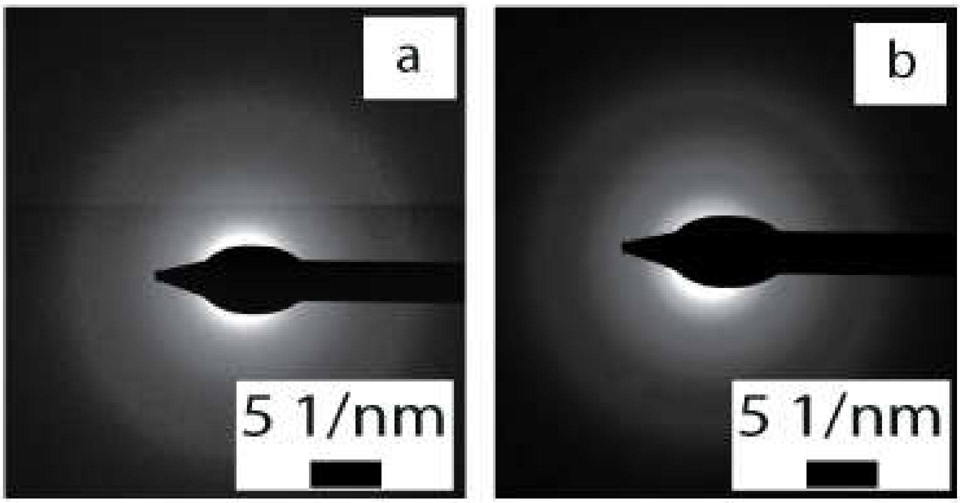

Figure S2. Selected area electron diffraction (SAED) of a) polystyrene phase containing MWCNT and b) polypropylene+copolymer phase in the blend PP:PS/70:30/MWCNT 1.0 vol.%/SB D1431P

3. SEM micrographs of PP:PS/70:30/MWCNT and PP:PS/70:30/MWCNT blends at different MWCNT concentration and constant 1.0 vol.% copolymer concentration.

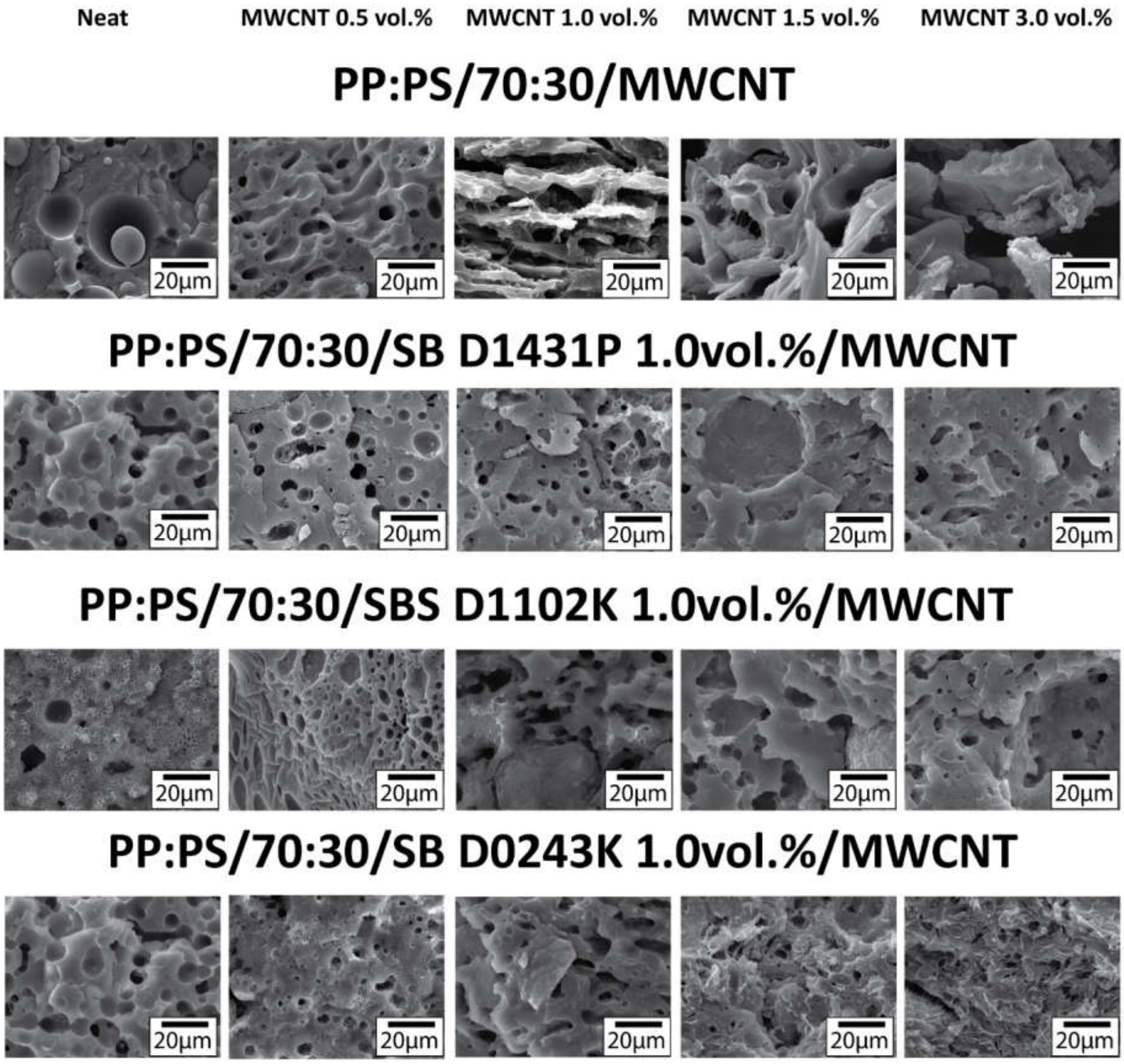

Figure S3. SEM micrographs of PP:PS/70:30/MWCNT and PP:PS/70:30/MWCNT blends at different MWCNT concentration and constant 1.0 vol.% copolymer concentration. From left to right MWCNT increases. From top to bottom: PP:PS/70:30/MWCNT, PP:PS/70:30/SB-D1431P 1.0 vol.%/MWCNT, PP:PS/70:30/SBS-D1102K 1.0 vol.%/MWCNT and PP:PS/70:30/SB-D0243K 1.0 vol.%/MWCNT.

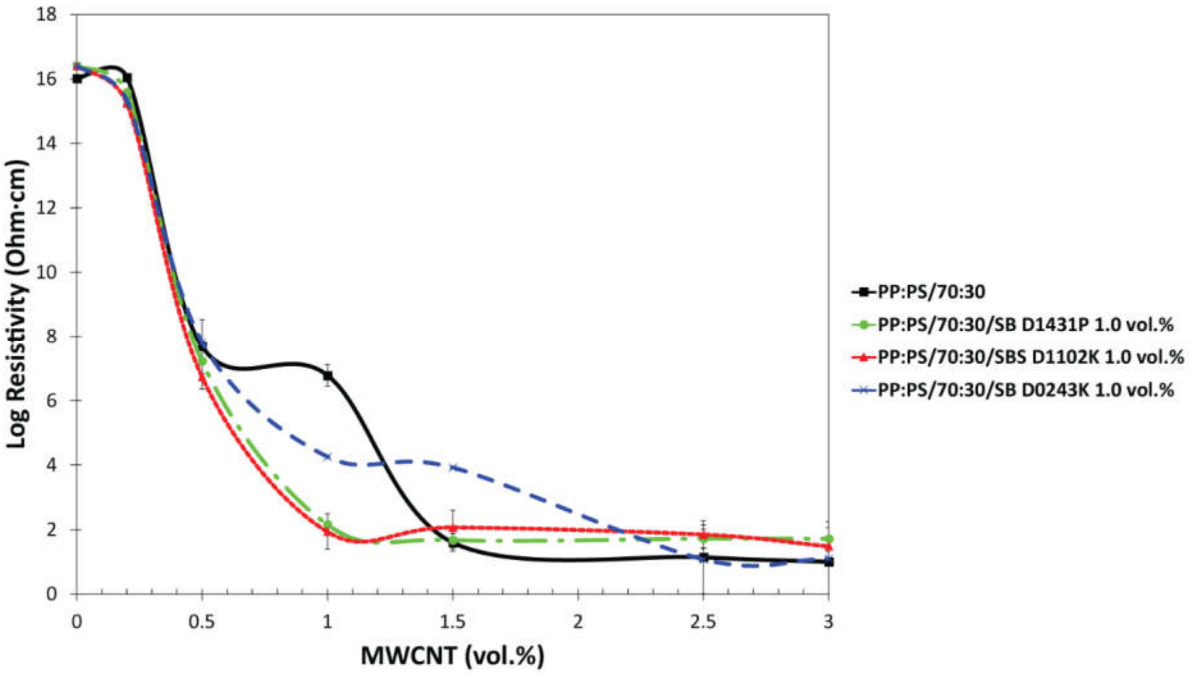

Figure S4. Percolation curves of PP:PS/70:30/Copolymer 1.0 vol.%.

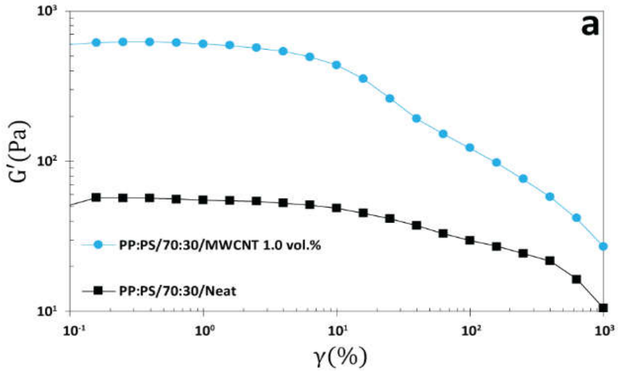

Figure S5. Oscillatory amplitude sweep results of PP:PS/70:30 without and with MWCNT 1.0 vol.%.
